# Evaluating Established Roles, Future Perspectives and Methodological Heterogeneity for Wilms’ Tumor 1 (WT1) Antigen Detection in Adult Renal Cell Carcinoma, Using a Novel N-Terminus Targeted Antibody (Clone WT49)

**DOI:** 10.3390/biomedicines10040912

**Published:** 2022-04-15

**Authors:** Dorin Novacescu, Talida Georgiana Cut, Alin Adrian Cumpanas, Silviu Constantin Latcu, Razvan Bardan, Ovidiu Ferician, Cosmin-Ciprian Secasan, Andrei Rusmir, Marius Raica

**Affiliations:** 1 Doctoral School, Victor Babes University of Medicine and Pharmacy Timisoara, E. Murgu Square, Nr. 2, 300041 Timisoara, Romania; dorin.novacescu@yahoo.com (D.N.); latcu_silviu@yahoo.com (S.C.L.); rusmirandrei@yahoo.com (A.R.); 2 Center for Ethics in Human Genetic Identifications, Victor Babes University of Medicine and Pharmacy Timisoara, E. Murgu Square, Nr. 2, 300041 Timisoara, Romania; 3 Department XIII, Discipline of Infectious Diseases, Victor Babes University of Medicine and Pharmacy Timisoara, E. Murgu Square, Nr. 2, 300041 Timisoara, Romania; 4 Department XV, Discipline of Urology, Victor Babes University of Medicine and Pharmacy Timisoara, E. Murgu Square, Nr. 2, 300041 Timisoara, Romania; razvan.bardan@gmail.com (R.B.); ovidiu.ferician@gmail.com (O.F.); cipriansecasan@gmail.com (C.-C.S.); 5 Department II, Discipline of Histology, Victor Babes University of Medicine and Pharmacy Timisoara, E. Murgu Square, Nr. 2, 300041 Timisoara, Romania; marius.raica@umft.ro; 6 Angiogenesis Research Center, Victor Babes University of Medicine and Pharmacy Timisoara, E. Murgu Square, Nr. 2, 300041 Timisoara, Romania

**Keywords:** adult renal cell carcinoma, Wilms’ tumor 1, WT1, clone WT49, immunohistochemistry, molecular pathology, diagnosis, prognosis, biomarker, novel therapeutic targets

## Abstract

Renal cell carcinoma (RCC) is arguably the deadliest form of genitourinary malignancy and is nowadays viewed as a heterogeneous series of cancers, with the same origin but fundamentally different metabolisms and clinical behaviors. Immunohistochemistry (IHC) is increasingly necessary for RCC subtyping and definitive diagnosis. *WT1* is a complex gene involved in carcinogenesis. To address reporting heterogeneity and WT1 IHC standardization, we used a recent N-terminus targeted monoclonal antibody (clone WT49) to evaluate WT1 protein expression in 56 adult RCC (aRCC) cases. This is the largest WT1 IHC investigation focusing exclusively on aRCCs and the first report on clone WT49 staining in aRCCs. We found seven (12.5%) positive cases, all clear cell RCCs, showing exclusively nuclear staining for WT1. We did not disregard cytoplasmic staining in any of the negative cases. Extratumoral fibroblasts, connecting tubules and intratumoral endothelial cells showed the same exclusively nuclear WT1 staining pattern. We reviewed WT1 expression patterns in aRCCs and the possible explanatory underlying metabolomics. For now, WT1 protein expression in aRCCs is insufficiently investigated, with significant discrepancies in the little data reported. Emerging WT1-targeted RCC immunotherapy will require adequate case selection and sustained efforts to standardize the quantification of tumor-associated antigens for aRCC and its many subtypes.

## 1. Introduction

With the advent of the widespread routine use of ultrasonography and computer tomography (CT) in clinical practice, the incidence of renal cell carcinoma (RCC) has shown a steady increase of 3% per year over the past 5 decades [1]. Conversely, the stage of disease at diagnosis has decreased, with most cases being incidentally discovered localized tumors, a subgroup of patients which has demonstrated improved 5-year survival rates [2]. However, confoundingly, the mortality rate for RCC per unit population, regardless of ethnic group or gender, has been steadily increasing since the 1980s, even though there are fewer advanced stages being diagnosed [3]. Thus, some aggravating occult modifications in tumor biology must have occurred over the past few decades, perhaps related to environmental factors (diet, tobacco use and exposure to other carcinogens) [2]. Today, RCC accounts for 2–3% of all adult cancers and is the most lethal common urologic cancer [3]; thus, imperatively mandating the identification of biomarkers able to better predict clinical outcome and therapeutic response. 

Traditionally, the term “RCC” defines a malignant neoplasm originating from the renal parenchyma (renal tubular epithelial cells), yet recent advances in genetic profiling and molecular analysis have nuanced this paradigm, as the current understanding of RCC refers to a heterogeneous group of distinct tumor subtypes, manifesting broad genomic pleomorphism, with subsequent variability in clinical behavior and therapeutic response [4,5]. Contemporary RCC subtyping is based on the 4th edition of the *Urological Tumors Classification*, published in 2016 by the World Health Organization (WHO), which identifies over a dozen types of malignant renal cell tumors, alongside mesenchymal, neuroendocrine, nephroblastic and cystic variants [4]. Already, additional entities have been recently described in the literature, based on distinguishing clinical, morphological, immunohistochemical and molecular characteristics, albeit still not formally recognized [6,7,8,9,10,11,12,13].

Microscopic evaluation of conventionally stained RCC specimens, although important, has become insufficient for differential diagnosis, in light of recent molecular insights into primary renal neoplasm biology. The multitude of emerging RCC entities, with their broad spectrum of poorly defined and highly disputed morphological characteristics, have made ancillary studies increasingly necessary for RCC subtyping and definitive diagnosis. Although classical histological variants of RCC manifest relatively characteristic microscopic features, the evaluation is user dependent, and morphologic similarities, at least focally, are easily encountered, thus hindering diagnosis. High-grade and poorly differentiated renal tumors are almost impossible to diagnose based on morphology alone, and this fact applies to metastatic sites, as well, with the additional difficulty of having to take into consideration tumors of non-renal origin [5]. Moreover, pathologists have reached the general consensus that, with minor exceptions—oncocytomas and some small (≤5 mm) low-grade papillary adenomas—there are no reliable histologic or ultrastructural criteria to differentiate benign from malignant renal epithelial tumors [14].

Even within conventional morphologies, absolute homogeneity is rare, with nonspecific arrangements of growth patterns (solid, papillary, cystic, tubular, sarcomatoid or rhabdoid) and cellular features (cytoplasmic clearing, eosinophilia or basophilia) usually being encountered, especially in high-grade or poorly preserved areas. Thus, meticulous sampling of gross specimens, in cases which are difficult to classify, may prove to be more useful and cost-effective than additional staining, as transitional areas, switching from well-differentiated low-grade patterns to more pleomorphic layouts, will most likely offer the most valuable diagnostic information [5,15].

Nowadays, definitive pathological diagnosis in surgical oncology, in general, requires careful integration of clinical workup and macroscopic and routine microscopic evaluation, alongside ancillary studies, most importantly immunohistochemistry (IHC), which, in recent years, has greatly added to diagnosis objectivity. Although a myriad of potentially useful diagnostic and prognostic applications for novel IHC antibodies in the pathological assessment of RCC have been described in recent years, this evaluation method is still imperfect, with multiple technical (clone selection, titration, validation, false positives/negatives, etc.) and interpretative (subcellular localization and pattern) deficiencies [15]. Regarding needle biopsies, cell blocks and fine-needle aspirates, there are limited data available on IHC antibody applications [16,17].

The *Wilms’ tumor 1 (WT1)* gene, mapping to chromosome 11p13, was initially described in 1990 as being a likely predisposing gene for nephroblastoma (Wilms’ tumor) [18,19], a pediatric kidney cancer affecting 1 in 10,000 children [20] and an archetypal model for tumorigenesis resulting from development gone awry [21]. Interestingly, although WT1 is, without a doubt, the most sensitive and relatively specific marker for the diagnosis of Wilms’ tumor, being positive in more than 90% of cases [22], only ~15% of nephroblastomas manifest *WT1* germline or somatic mutations [20]. In fact, Wilms’ tumor is a primarily sporadic disease, as only 1–2% of cases have a positive family history [23]. Even so, recurrent loss-of-function mutations in multiple tumor-suppressor genes (i.e., *WT1, p53, familial WT1/2 (FWT1/2)* and at the *11p15.5 locus* [24]) have been reported.

In mammals, the *WT1* gene has a length of ∼50 kilobases and 10 encoding exons, resulting in a variety of potential WT1 isoforms (at least 36), due to subsequent alternative transcription/translation start sites, splicing and RNA editing [22]. As a common denominator, similarly to SP1-transcription factors, all isoforms include four zinc fingers [22], which are able to activate or repress other target genes involved in controlling cell growth and development [25,26,27], manifesting a multitude of complex, occasionally opposing functions, ranging from organogenesis, tissue homeostasis maintenance and tumor suppression to oncogenesis [23,27,28,29,30,31,32,33].

On the one hand, *WT1* behaves as a classic tumor-suppressor gene, requiring both alleles to be deleted or inactivated for tumor development to occur [34]. However, other mechanisms may also be at play, as a majority of tumors manifesting *WT1* mutations also have gain-of-function mutations in the *ß-catenin (CTNNB1)* gene and, additionally, due to loss of heterozygosity in chromosome 11, *insulin-like growth factor 2 (IGF2)* overexpression [35]. On the other hand, WT1 is expressed in a wide range of adult tumor subtypes of epithelial, mesenchymal, hematopoietic and neuronal origin, without being expressed in the corresponding healthy tissue, thus mandating the proposition that *WT1* functions as an oncogene in these situations. Even though, currently, the evidence supporting this claim is limited [23,31], the wide spectrum of tumors manifesting WT1 expression has made it one of the most promising targets in cancer immunotherapy [36].

WT1 mRNA seemingly displays a developmental and tissue restricted pattern of expression, being highest during urogenital embryogenesis [37]. Thus, WT1 is firstly identified in the genitourinary ridge, well expressed in both pronephric and mesonephric structures. In contrast, the metanephric blastema initially manifests scarce WT1 expression, which is then accentuated during condensation into S-shaped bodies. As subsequent differentiation progresses, WT1 expression will increase in the glomerular epithelial cells and will be lost in the differentiated tubules [38]. Conversely, nephroblastomas apparently manifest most of the elements found in the normal embryonic kidney, but in a disorganized state; however, in contrast to their normal counterparts, the tubular epithelial-like structures in nephroblastomas frequently express WT1 transcripts [39]. This alteration of the normally restricted pattern of expression of WT1 may be reflective of an underlying cellular de-differentiation process, with mesenchymal-derived kidney cells transforming into more immature blastema-like cells [38]. 

Broadly believed to originate from the proximal convoluted tubule, aRCCs have been previously shown to constitutively express substantial amounts of WT1 RNA and protein, albeit in a small subset of cases [38]. Moreover, DNA sequencing, nuclear location and size of the WT1 protein, coupled with its ability to interact with p53, indicate that RCCs appear to express normal WT1 protein, meaning that WT1 acts as a transcriptional regulator in RCCs and may contribute to the tumorigenic phenotype [38]. This being said, the intuitive retrograde approach, based on modulating the expression of WT1 in RCC, is being evaluated in multiple experimental and clinical applications (WT1-targeted vaccination with peptide, mRNA or loaded dendritic cells [40,41]; WT1-targeted cytotoxic T lymphocytes, obtained by using induced pluripotent stem cell technology [42]), which have shown promising initial results in suppressing RCC tumor phenotypes.

Currently, WT1 IHC staining is mainly used in the pediatric setting in order to distinguish between Wilms’ tumor and other pediatric renal tumor subtypes, due to its aforementioned high sensitivity for its namesake tumor. Still, the method is imperfect, and diagnostic pitfalls linger, seeing as specificity is lacking, with WT1 also being expressed in morphological mimics of nephroblastoma. Moreover, variable staining patterns have been documented, depending on the type of antibody used (C-terminal vs. N-terminal) [22].

Wilms’ tumor, a malignant pediatric embryonal neoplasm, the result of a cellular differentiation disturbance at the level of the metanephric blastema [43], replicates, at least partially, the morphology of the developing metanephros, being the most common genitourinary tumor of childhood (peak incidence between 2 and 4 years of age). Frequently, bilateral renal involvement can be encountered (in 5–10% of cases, involvement is synchronous/metachronous) [22].

Most Wilms’ tumors usually manifest a characteristic triphasic histological layout comprising the following components:

- Blastematous: diffuse, nodular and/or cord-like growth patterns of small, round and huddled blue cells, showing dark nuclei, diminished cytoplasm and frequent mitosis);
- Epithelial (tubules, papillae and glomeruli, similar to structures found in normal nephrogenesis, alongside more primitive rosette-shaped early tubular structures, and cysts lined with immature columnar/cuboidal cells);
- Stromal (resembling embryonal mesenchyme, spindle-shaped cells are seen on a myxoid stromal background, with a variety of patterns of differentiation and heterologous stromal elements, most commonly skeletal muscle in various stages of differentiation, including rhabdomyoblasts, but also cartilage, bone, fat or, more rarely, neuroglia and mature ganglion cells) [22,43].

The IHC profile depends on which tumor component is being examined. Blastematous components are usually diffusely positive for vimentin and WT1, with variable staining patterns having been described for CD56/57, cytokeratins (CK)s, epithelial membrane antigen (EMA), desmin and paired box (PAX)2 [37,44,45,46,47,48]. Epithelial elements are usually positive for CKs, EMA, CD56 and, with variations, PAX2 and WT1 [44,45,46]. The stromal component is usually stains for vimentin, while the heterologous skeletal muscle, when present, will be identified by positive staining for desmin, myogenin and MyoD1 [22].

Pediatric diagnostic difficulties are centered around Wilms’ tumor cases comprising almost exclusively the aforementioned blastematous component, especially when dealing with small biopsy samples (differential diagnosis with desmoplastic small round cell (DSRC) tumor, malignant rhabdoid tumor, rhabdomyosarcoma and neuroblastic tumors). Staining for WT1 should be performed with both types of WT1 antibodies (C-/N-terminus), while additional IHC markers (INI1, useful in ruling out malignant rhabdoid tumor) and additional molecular testing (reverse transcription polymerase chain reaction for EWS-WT1 novel fusion transcript, due to a recurrent chromosomal translocation t(11;22)(p13;q12), which is seen in ~90% of DSRC tumors [49]) will serve to further objectify definitive diagnosis [22].

In adults, WT1 IHC staining addresses the differential diagnosis conundrum of subtyping renal tumors with a significant papillary component. A subpopulation of type 1 papillary(p) RCCs, characterized by a mostly solid pattern of growth, may be misconstrued as metanephric adenoma (one of the rarest benign renal tumors [50]) or even highly differentiated epithelial-predominant Wilms’ tumor [5]. Precisely because Wilms’ tumor rarely occurs in adults, with only 3% of cases being reported in patients >16 years old, it is frequently misdiagnosed as metanephric adenoma [51], since both entities are positive for WT1, with serious implications regarding prognosis and therapeutic strategy [52,53]. Although meticulous evaluation of nuclear details may help solve this differential diagnosis, an IHC panel that includes CK7, racemase(AMACR), WT1 and CD57 is also useful for definitive diagnosis [54], with special attention being paid to tumor transitional areas (low grade to high grade cellularity). Solid pRCC will be positive for CK7 and AMACR, but negative for WT1 and CD57, while, conversely, metanephric adenoma is only positive for WT1 and CD57, whereas Wilms’ tumor expresses only nuclear immunoreactivity for WT1 and, rarely, isolated cells with CK7 cytoplasmic positivity. Moreover, within the same papillary component subgroup, carbonic anhydrase IX (CAIX), CK7 and AMACR staining can distinguish between clear cell (cc)RCC with papillary areas and true pRCC and/or clear-cell-papillary (ccp) RCC [5]. 

In the metastatic context, the main diagnostic valuable of IHC results in determining primary tumor origin resides in positive staining with high specificity markers. However, for most markers, the evaluation of staining results in a binary fashion (i.e., positive and negative) has proven to be very difficult (due to focal patch staining, lacking clear cutoff criteria), and potentially misleading, mandating the integration of both quantitative staining estimations and marker expression pattern analysis [18]. In the case of metastatic RCC (mRCC), for any given marker, sensitivity and specificity are dependent upon multiple factors: specific antibody clone and method of detection used, size and quality of specimen analyzed (abundant necrosis, small core biopsy or fine-needle aspiration specimens can affect sensitivity of focal labeling markers) and RCC grade (better labeling in more differentiated low-grade RCCs) [5]. Rarely, RCCs may metastasize to the pleura and lung and, due to the wide variety of morphological patterns for both mesothelioma and RCC, a misdiagnosis may occur. A previous investigation proposed calretinin, mesothelin and cytokeratin(CK)5/6 as being the best positive mesothelioma markers for differentiating epithelioid mesotheliomas from RCCs, while the best discriminators for mesothelioma were CD15, MOC-31 and RCC marker antigen (RCCm). WT1 had also been investigated, but appeared to be less sensitive and somewhat less specific than the three previously mentioned positive markers, seeing as WT1 reactivity was seen in 93% of the epithelioid mesotheliomas evaluated, but also in a single case of ccRCC that had metastasized to the lung (out of 40 evaluated) [55].

With the advent of promising novel WT1-targeted immunotherapy modalities for aRCCs, adequate patient selection criteria will be essential in order to correctly personalize treatment and not waste valuable resources. More extensive studies into the metabolomics of aRCCs, especially regarding the molecular dynamics/implications of WT1 protein expression modulation, are mandatory for a better understanding of how to evaluate the possible benefits/pitfalls of these novel therapeutic tools. In fact, beyond nephroblastomas, the currently available data on the topic of evaluating WT1 expression by using IHC in aRCCs are scarce and inconsistent. The few relevant investigations used different antibodies and IHC protocols, yielding various nuclear/cytoplasmic staining patterns, as well as different interpretation methodologies, regarding positivity definitions and quantitative/qualitative quantifications. A novel monoclonal N-terminus targeted WT1 antibody, clone WT49, has recently become commercially available and has not yet been investigated, to the best of our knowledge, in aRCCs, nor in pediatric RCCs for that matter.

For these reasons, we found it useful to evaluate, in the current study, the expression of WT1 protein, using WT1-targeted IHC (N-terminus, monoclonal antibody and clone WT49), in a consecutive single-center series of aRCC cases, in an attempt to investigate, for the first time, staining patterns of a novel WT1 antibody in aRCCs. In the largest investigation to date, focusing exclusively on aRCCs, we found an exclusively nuclear pattern for WT1 immunoreactivity in all positive cells. We also report the highest rate of WT1 nuclear staining for aRCCs to date. To integrate these findings, we review and address methodological heterogeneity and variability in reporting regarding RCC WT1-targeted IHC in order to reconcile discrepancies and standardize evaluation of WT1 antigen expression, with the hope of better understanding the usefulness and possible clinical applications of this method in the management of aRCCs.

## 2. Materials and Methods

In this study, we aimed to retrospectively evaluate the IHC expression of WT1 antigen in adult renal cell carcinoma (aRCC). To this end, after obtaining adequate approval from our Institutional Review Board (26/28 September 2018), we have analyzed a recent (2016/2017) consecutive series of 90 cases of aRCC from the Arad County Hospital, Urology and Morphopathology Department, which all underwent radical nephrectomy per primam, without any neoadjuvant treatment. Paraffin-embedded post-radical nephrectomy tumor specimens were obtained. A microtome was used to obtain multiple 3 μm–thick sections for each case. These sections were further processed by being positioned on albumin-treated silanized tissue glass slides and stretched out in a liquid environment (one drop of distilled water is added) in order to avoid artefact formation. After the excess distilled water was removed, the slide was thermally treated (thermostat, 58 degrees Celsius, for a minimum of 30 min).

After a preliminary deparaffination stage (benzene, 58 degrees Celsius, for 30 min), the tissue slides were stained with Hematoxylin–Eosin (HE), in an automated manner, using the Leica Autostainer XL (Leica Biosystem Newcastle Ltd., Balliol Business Park West, Benton Lane, New Castle Upon Tyne, UK), following the standardized protocol. 

After HE staining, all the tissue slides were evaluated by using a Nikon E600 photon microscope. The main inclusion criteria for WT1 IHC staining was represented by the presence of healthy renal tissue in the immediate proximity of the tumor on HE staining, seeing as the podocyte expression of WT1 in normal renal corpuscles will represent the internal positive control reference. 

After the inclusion criteria were met, samples were further classified based on the predominant morphological pattern and nuclear traits, using the modified Fuhrman grading system (Table 1). Tumor stroma and the presence of inflammatory infiltrate were crudely evaluated microscopically in the HE-stained samples, and tumors which had massive inflammatory infiltrates in the tumor stroma were noted as positive.

These samples then underwent IHC staining. WT1 (monoclonal, clone WT 49, N-terminus targeted, ready to use, from Leica Biosystem, Newcastle Ltd., Newcastle Upon Tyne, UK) was use as primary antibody, pre-diluted (30 min, room temperature). Heat-induced epitope retrieval with Bond Epitope Retrieval Solution 2 (Leica Biosystems, Newcastle Ltd., Newcastle upon Tyne, UK) for 20 min was followed by endogenous peroxidase blocking (5 min), incubation with primary antibodies (20 min) and visualization with The Bond Polymer Refine Detection System (15 min). The 3,3-diaminobenzidine dyhidrochloride was used as chromogen (10 min), and hematoxylin was used as a counterstain. The full immunohistochemical procedure was performed with Bond Max autostainer (Leica Biosystem).

After WT1 staining, all slides were scanned by using Desk Pannoramic Scanner (3D Histech, Budapest, Hungary), and they were stored on Histology Department Digital Slides Library Case Center). The evaluation of the slides, pictures capture, and processing were performed by using Pannoramic Viewer program (3D Histech, Budapest, Hungary). For each sample, the quantitative immunoreactivity of the tumor tissue for WT1 was established, following the protocol described in Table 2. Additionally, qualitative labels were attributed, by comparison with internal positive control (podocytes), regarding color intensity and homogeneity: weak, moderate and high.

## 3. Results

We found 56 viable cases which met the inclusion criteria.

**Figure 1:**
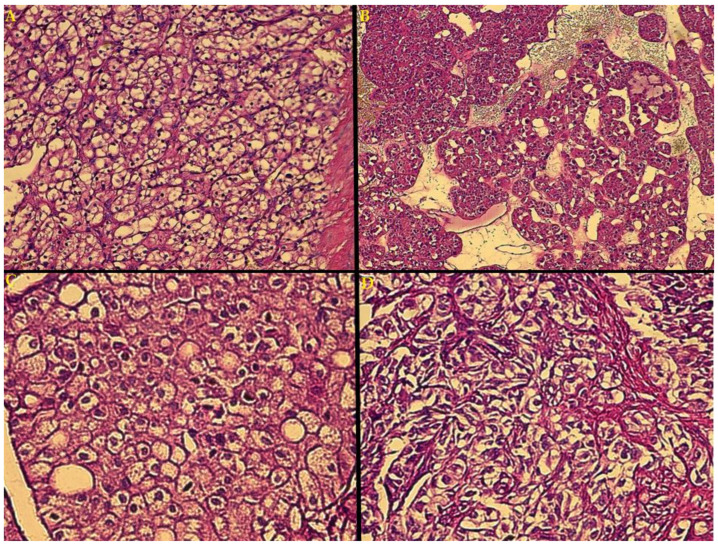
**HE staining**: (**A**) 200×, conventional solid clear cell renal carcinoma; (**B**) 200×, papillary architecture of RCC; (**C**) 400×, chromophobe RCC—clear cytoplasm with chromophobic perinuclear halo; (**D**) 200×, sarcomatoid dedifferentiation.

**Figure 2:**
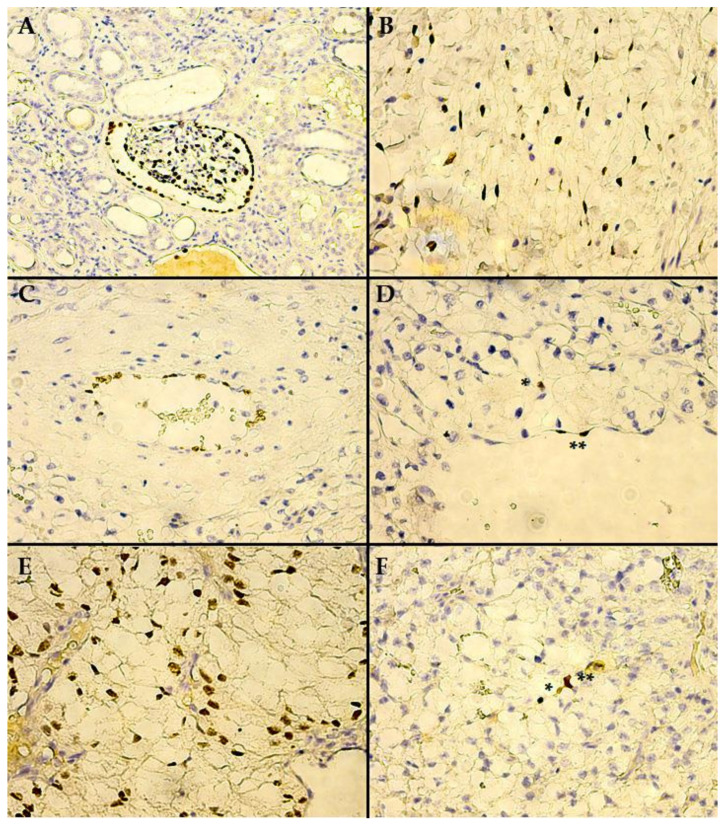
**WT1 IHC staining**: (**A**) 200×, control image of a quasi-normal positive glomerulus; (**B**) 400×, positive fusiform cells outside of the tumor tissue, with a morphology suggestive of fibroblasts/myofibroblasts; (**C**) 400×, blood vessel with a majority of endothelial cells manifesting positive nuclear staining; (**D**) 400×, large blood vessel close-up, with scarce positive endothelial cells (**) and a single positive adjacent tumor cell (*); (**E**) 400×, moderate density of positive tumor cells (+2), manifesting predominantly high intensity nuclear staining; (**F**) 400×, low density (+1) tumor tissue, manifesting only 2 distinct positive cells, with weak-intensity (*) and moderate-intensity (**) nuclear staining.

**Figure 3:**
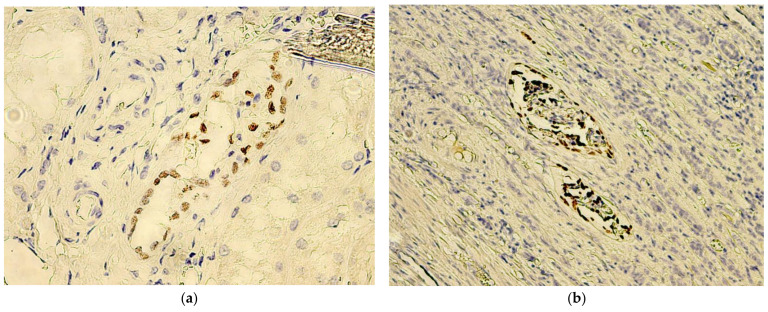
**WT1 IHC staining**: (**a**) 400×, positive connecting tubule cells, low-to-moderate nuclear staining; (**b**) 200×, positive renal corpuscles with severe degenerative lesions, compressed by neighboring tumor tissue, but with relatively unaffected podocytes.

Based on predominant morphology, four case groups were identified: clear cell (Figure 1A)—38, papillary growth (Figure 1B)—8, chromophobe architecture (Figure 1C)—3 and sarcomatoid growth (Figure 1D)—7. Nuclear grading further classified these tumors: G1—21, G2—23, G3—4 and G4—8.

The normal renal tissue adjacent to the tumor constantly manifested for all of the evaluated cases, nuclear WT1 staining of podocytes and, also, of the epithelial cells, constituting the parietal layer of Bowman’s capsule (Figure 2A). The final reaction product steadily displayed an intense brown coloring for the aforementioned positive internal control, while all of the components of the cortical tubular system were negative, with the exception of occasional (only two cases, with negative tumor tissue WT1 staining) low-to-moderate intensity nuclear staining of connecting tubule cells (Figure 3a). It is worth mentioning that the podocytes maintained intense immunoreactivity, even in the case of renal corpuscles, which otherwise showed obvious morphological signs of degeneration (Figure 3b), being, apparently, the most resilient cell line involved in the renal blood-filtration barrier.

In the renal capsule, but not in the tumor pseudocapsule, we identified numerous intensely positive stromal cells, which, taking into account cell morphology, are most likely fibroblasts and myofibroblasts. These types of cells were noted in 5 (8.92%) of the 56 evaluated cases, in the extrarenal connective tissue, located at a significant distance from the tumor tissue (Figure 2B). Noteworthy is the fact that, in all five cases, the tumor tissue was negative for WT1 staining.

Another encountered particularity, referring strictly to intratumoral endothelial cells, is the fact that, in 3 of the 56 cases analyzed, these cells manifested positive WT1 nuclear staining, while tumor cell immunoreactivity for WT1 was negative for all of the three cases. Two cases showed mid-size arterial structures, demonstrating a majority of positive endothelial cells (Figure 2C), while, in the third case, a large vein manifested scarce positive endothelial cells, with a single adjacent positive tumor cell (Figure 2D)—the case was not considered to be a WT1 positive tumor, seeing as it did not meet the minimum set of quantitative analysis criteria described in Table 2. 

Interestingly, all three cases of WT1-negative tumors with WT1-positive intratumoral endothelial cells demonstrated significant stromal inflammatory infiltrate, in relatively close proximity to the positive vascular structures, upon HE evaluation, but with no WT1 immunoreactivity documented in the set infiltrates.

**Figure 4:**
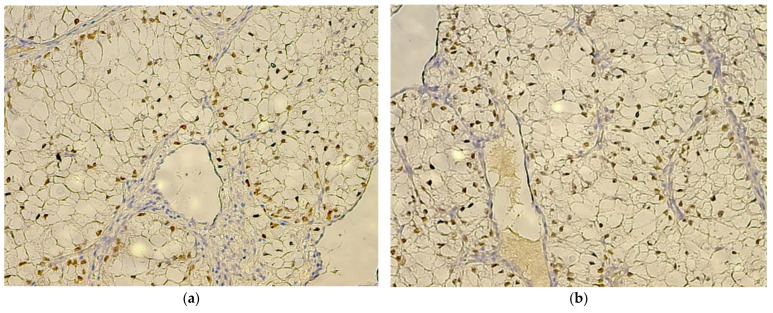
**High-density clear cell nuclear WT1 IHC staining (+3), moderate intensity.** (**a**) Case 1: 200×. (**b**) Case 2: 200×.

Out of the 56 cases evaluated, 49 demonstrated negative WT1 staining within the tumor tissue. The seven positive cases (see Table 3) all manifested intratumoral, exclusively nuclear, WT1 staining, and all had a clear cell morphology. The quantitative analysis revealed that only two cases had a high density of positive tumor cells (+3) (Figure 4a,b). The intensity of staining for the final reaction product from within the positive tumor cells was similar to the control (podocytes) or higher (Figure 2E), except for the two cases with high positive tumor cell density, which manifested a lower intensity than the control. Interestingly, one of the positive cases, which was labeled +1, showed only two tumor cells with nuclear staining for WT1 (Figure 2F), with weak and moderate intensity, respectively.

Interestingly, although more than a quarter of evaluated cases showed massive inflammatory infiltrates, visibly on HE-stained samples, the WT1-positive tumors showed very little to no morphological infiltration of immune cells into the tumor stroma. Moreover, none of the tumors which had abundant inflammatory infiltrate manifested WT-1 positivity, not at the level of tumor cells, nor at the level of the immune cells invading the tumor stroma.

Noteworthy is the fact that, although we used an N-terminus targeted monoclonal antibody (clone WT49), no cytoplasmic staining was encountered in the tumor specimens evaluated.

Taking into account the inherent limitations of our study’s design (observational and retrospective) and the limited number of cases evaluated, no statistically significant conclusions can be drawn regarding correlations between WT1 intratumoral expression and tumor aggressiveness (histology, local extension, nuclear grade and overall survival/recurrence rate).

## 4. Discussion

RCC is the deadliest of all urological malignancies [56], despite sustained efforts to improve treatment modalities and, implicitly, patient outcomes. Nowadays, RCC is a term that is used to define a heterogeneous series of cancers which have a common origin in the renal tubular cells, yet are driven by distinct genetic injuries and signaling pathways [57,58]. Naturally, each RCC subtype will, therefore, manifest different pathological features and variable dissemination patterns [59], accounting for the modest success levels in treating mRCC achieved so far [60]. Although IHC expression of WT1 is usually undetectable in renal epithelial structures originating from the metanephric mesenchyme, i.e., proximal/distal tubules, WT1 transcripts have been documented in tubular structures within nephroblastomas [38]. Therefore, the investigation of WT1 protein levels in aRCCs seems intuitive, yet very few studies on the topic are available, and the results are inconsistent. Thus, we found it necessary to elaborate this study, which is, currently, to the best of our knowledge, the largest aRCC-dedicated tumor tissue WT1 protein expression analysis.

The first and only other dedicated investigation, undergone over 20 years ago, on the topic of constitutive expression of WT1 in aRCCs used cell cultures (five ccRCCs and one oncocytoma) and demonstrated that four out of five ccRCCs and the only oncocytoma tested expressed measurable WT1 RNA, at levels less than those observed in the human fetal kidney cultures (included for comparison), but significantly higher than those seen in cultured proximal tubular epithelial cells. Cytochemical immunoreactivity of RCC cryosections showed ample WT1 protein levels within undifferentiated tumor areas comprising epithelial components. RCC immunoblots detected WT1 protein within the normal size range. RNase protection analysis showed a relative ratio of alternative splice variants (four transcripts) between the different RCC samples, similar to those seen in the fetal kidney. Moreover, RCC cell extract immunoprecipitations indicated that WT1 interacts, in this context, with p53, a relationship already reported in the healthy kidneys of human fetuses. Thus, it may be the case that the abnormal RCC levels of functional WT1 protein documented may actually be indicative of a cellular dedifferentiation process, which may very well contribute to the tumorigenesis by consequent transactivation of specific target genes involved in cell growth regulation [38].

The rest of the available data on WT1 protein expression in aRCCs come from comparative studies with other types of neoplasms, mainly pleural mesothelioma and nephroblastoma, known for consistent WT1 nuclear staining, and the contradictions are rampant. An early study comparatively evaluated WT1 protein levels, using three non-commercially available monoclonal antibodies, in malignant mesotheliomas, as opposed to primary lung tumors and metastatic pleural lesions. The single mRCC case evaluated showed positive nuclear staining [61]. One author evaluated IHC expression of TTF-1, E-Cadherin, BG8, WT1 (commercially available polyclonal antibody) and CD44S in pleural mesothelioma, as compared to pulmonary and non-pulmonary adenocarcinomas, of which 10 RCCs (five primary and five metastatic to the lung), and found all RCC cases were negative for WT1 nuclear staining [62]. The same author, in a later investigation, evaluated a different, wider panel of IHC markers, again including WT1 (6F-H2 monoclonal antibody), in 48 mesotheliomas (40 epithelioid/8 sarcomatoid), and the same number of RCCs (24 clear cell, 12 chromophobe, 8 papillary and 4 sarcomatoid), and found WT1 nuclear expression in >50% tumor cells in a single case of conventional ccRCC that had metastasized to the lung (4% of the ccRCC cases evaluated) [55]. A few years before, a different investigation, using the same 6F-H2 monoclonal antibody for WT1, documented focal nuclear reactivity (<10% of the cells) in two out of the five chRCCs, but in none of the five ccRCCs and five pRCCs included in this study [63]. Lastly, the 6F-H2 monoclonal WT1 antibody was used, among other markers, to try to distinguish between tumors with clear cell morphology, namely female genital tract carcinomas (12 cases with ovarian and endometrial origin), ccRCCs (23 cases) and MiT-translocation associated RCCs (5 cases). No nuclear WT1 expression was seen in any of the RCCs analyzed [64].

More recently, a study investigating the diagnostic utility of WT1 immunostaining in pediatric renal tumors used the 6F-H2 monoclonal antibody on 53 pediatric cases of renal tumors (38 nephroblastomas, 6 mesoblastic nephromas, 2 clear cell sarcomas, 2 ccRCCs, 2 peripheral neuroectodermal tumors, 1 AML, 1 rhabdomyosarcoma and 1 malignant rhabdoid tumor). Out of the non-nephroblastoma population, both cases of ccRCC analyzed (mean age 11 years) showed weak-to-moderate (<50% of cells) diffuse cytoplasmic WT1 positivity, as well as strong (>50% of cells) cytoplasmic WT1 positivity for the one case of rhabdomyosarcoma (2-year-old), and moderate cytoplasmic WT1 staining for the one case of malignant rhabdoid tumor (2-year-old) [65]. These findings are consistent with a later investigation into pediatric renal tumor differential diagnosis, using both C- and N-terminus targeted WT1 antibodies, which concluded that, although useful for diagnosis of malignant tumors in children and adolescents, WT1 protein expression (nuclear and/or cytoplasmic, depending on the tumor and antibodies used) is not exclusive to nephroblastoma, being also encountered in DSRC and malignant rhabdoid tumors [22].

The emerging discussion, centered on the patterns of staining for WT1 (nuclear or cytoplasmatic), depending on the antibody used (C-terminus or N-terminus), is an important one, as it would partially explain the wide variability of reported results on aRCC WT1 staining. Traditionally, the IHC expression of WT1 protein, using older polyclonal C-terminus antibodies (clone WT 1C19), was considered to be exclusively limited to the nucleus, while any rarely encountered cytoplasmic staining was disregarded as a mere artefact (cross-reactivity). With the newer monoclonal N-terminus targeted antibodies (clone WT6F-H2), consistent WT1 staining has been obtained in the nucleus, cytoplasm or concurrently in both [66,67,68,69,70,71,72]. Beyond the antibody used, the variable immunoreactivity can be explained, on a molecular level, by recent investigations proposing WT1 as a regulator of transcriptional/translational processes, shuttling between the nucleus and the cytoplasm [27,73].

A large relatively recent investigation of WT1 protein expression used both polyclonal (C-19) and monoclonal (6F-H2) IHC antibodies on 494 human neoplasms of diverse origins (gastrointestinal/pancreatobiliary system, male/female urogenital system, mamary, pulmonary, cerebral, skin, soft tissues and bone). All cases with either nuclear or cytoplasmic staining for WT1 were considered positive. For the 15 RCCs evaluated (13 ccRCCs, 1 sarcomatous RCC and 1 pRCC), percentages regarding positively stained specimens varied, depending on the antibody used. As a whole, 47% of RCCs were positive for WT1, using C-19, with only 36% positivity using 6F-H2. Differences persisted within ccRCC cohort stratification: Fuhrman grade 1 (50% for C-19/25% for 6F-H2), grade 2 (50% for C-19/33% for 6F-H2) and grade 3 ccRCC (100% for both, but only one case). The sarcomatous RCC was negative for WT1 using both antibodies, but the pRCC case showed WT1 positivity only when using the 6F-H2 clone (negative for C-19) [74]. Since, for all RCCs evaluated, WT1 staining was exclusively cytoplasmic, the authors attribute the relatively high rate of positivity and discrepancies compared to previous data to the criteria used for the definition of WT1 positivity (exclusively nuclear versus nuclear/cytoplasmic staining). Moreover, the Western blot analysis performed within this investigation revealed that WT1 protein had mainly cytoplasmic expressions in lung adenocarcinoma cells (only two cases investigated). Even so, confoundingly, WT1 is fundamentally a 4-zinc-finger-motif transcription factor, with DNA/RNA binding abilities (C-terminus) [75], and it is also involved in transcriptional regulation, self-association and RNA recognition (proline/glutamine-rich N-terminus) [76,77,78], being mainly distributed within the nucleus.

In this study, we evaluated the expression of WT1 protein in 56 cases of aRCC (38 ccRCCs, 8 pRCCs, 3 chRCCs and 7 sarcomatoid RCCs), using a recently available N-terminus targeted monoclonal antibody (clone WT49), and we found seven positive cases (12.5%), all ccRCCs, showing nuclear staining for WT1. Regarding nuclear grading, three positive cases were Fuhrman grade 1, and the rest (four cases) were Fuhrman grade 2, meaning 14.28% of the total grade 1 cases and 17.39% of the grade 2. All pRCCs, chRCCs and sarcomatoid RCCs were negative for WT1, as well as all Fuhrman grade 3 and 4 cases. Curiously, even though we used an N-terminus targeted antibody and included cytoplasmic staining in the definition of positivity for WT1 immunoreactivity, none of the WT1-positive aRCCs manifested cytoplasmic staining, nor did we disregard cytoplasmic staining in any of the negative cases. These findings represent, to the best of our knowledge, the highest percentage of nuclear WT1 positive RCCs reported. Moreover, this is the first time this particular antibody (clone WT49) has been used to evaluate aRCCs. 

Quantitatively, only two cases (Fuhrman grade 1 and grade 2, respectively) had >10% (+3) WT1-positive cells, with three cases (two Fuhrman grade 1 and one grade 2) having ≥2%, but <10% (+2) WT1-positive cells, and two cases (both Fuhrman grade 2) having <2% (+1) WT1-positive cells. One of the +1 labeled positive cases actually had only two WT1-positive tumor cells (nuclear staining), with weak and moderate stain intensity, respectively. Qualitatively, the +3 high positive cell density tumors manifested moderate staining intensity, as compared to the podocyte control reaction, whereas all the +2 cases and the one other +1 case manifested high-intensity staining. Therefore, the extent of cellular WT1 positivity and/or the quality of WT1 staining seemingly do not correlate with Fuhrman nuclear grading scores, the main current indicator of aggressive cellularity in RCC. Moreover, TNM staging also did not seem to relate to WT1 protein expression, seeing as most positive cases were pT1a/b (localized tumors, <7 cm) and the only pT3a (invasion of renal sinus fat) WT1positive tumor had <10% of tumor cells positive for WT1. 

Overall survival and recurrence rates also did not correlate well with WT1 expression. The two cases with the most abundant WT1-positive cell rate (+3, >10%), a pT1b grade 1 and a pT2a grade 2, respectively, both with moderate intensity WT1 staining, were alive and had no documented recurrence at 5 years. Conversely, the two cases with the lowest rate of WT1-positive cell rate (+1, <2%), a pT2b and a pT1b, both grade 2, showing weak and moderate stain intensity of only two distinct cells for pT2b and high intensity staining for pT1b, were both dead at five years. The pT2b case, which was barely positive, died 3 years post radical nephrectomy, due to systemic dissemination (pulmonary metastases), whereas the 74-year-old pT1b case died of myocardial infarction and had no documented recurrence at 4 years. The +2 cases (<10%, but >2%) were all alive at 5 years, with only the pT3a case experiencing a recurrence after 2 and a half years (solitary lung metastasis) that was successfully treated, and no further recurrences were documented at 5 years.

Obviously, from a conceptual standpoint, when dealing with a complex gene that has a myriad of transcript isoforms, such as WT1, one must take into account the fact that IHC is a semi-quantitative investigation at best. It merely offers an incomplete singular snapshot of protein expression in the context of a dynamic, fluid and rapidly changing series of molecular events, constituting the biological microenvironment of RCCs. Therefore, a priori, few conclusions, if any, can be drawn by using this method alone, regarding the actual molecular role of the *WT1* gene in RCC tumorigenesis and aggressiveness.

This being said, the *WT1* gene and its many transcript isoforms represent a very good example of the intrinsic complexity and apparently endless variability of molecular signaling mechanisms, as distinct proteic transcripts, with minor, apparently insignificant differences in amino acid sequences, manifest completely divergent functions. Even though our understanding of *WT1*-mutation-related tumorigenesis is incomplete, over the past decades, analyses of this topic have provided significant knowledge regarding the following:

- The development, homeostasis and pathology of intermediate and lateral plate mesoderm derived tissues;
- Transitional cellular events during development, i.e., mesenchyme-to-epithelial transitions (MET)/epithelial-to-mesenchyme transition (EMT);
- Mesenchymal progenitors’ cellular origin in a wide array of human tissues, highlighting the essential role of the mesothelium as a mesenchymal progenitor source;
- Essential inner workings of transcription and epigenetic regulation [25].

Overall, phenotypic modifications caused by *WT1* mutations correlate well with the gene’s expression patterns, as reported during fetal development. *WT1 null mice* will be unable to develop kidneys and gonads; will exhibit congenital diaphragmatic hernia; and will die in utero, on average, at ~13.5 embryonic days, presumably due to cardiac problems [79], while also manifesting hypoplastic lungs, splenic agenesis [80] and adrenal gland agenesis [81,82].

In humans, alternative splicing for *WT1* mainly occurs in two distinct regions: a mammalian-specific splicing site, within the central region of the protein, with a 17 aminoacid insertion (17AA); and a non-mammalian vertebrate-specific site, within the zinc finger region, with a lysine–threonine–serine (KTS) sequence insertion between zinc fingers 3 and 4. Even though an individual functional relevance for this myriad of WT1 isoforms has yet to established, mutations in the KTS splice site have been documented in patients with Frasier syndrome [83], meaning gonadal dysgenesis, ambiguity to complete transformation of male external genitalia to a female phenotype and progressive glomerulonephropathy (focal segmental glomerulosclerosis). 

Apparently, both KTS-positive (+) and KTS-negative (−) WT1 transcripts are crucial and manifest distinct functions. Frasier syndrome genomics (due to *WT1* mutations, only the transcription of KTS− isoforms can occur [25]), but also KTS gene targeting experiments in transgenic mice support this idea, as deficiency in either isoform (+/−) results in perinatal demise of the animal model, due to incomplete renal development. Noteworthy, deficiency of KTS− isoforms determines more severe phenotype deteriorations than KTS+ isoform deficit [84]. Important from an IHC standpoint, KTS− isoforms, with the zinc finger domain, corresponding to the C-terminus of protein transcripts and lacking KTS insertion, will have a much more pronounced DNA affinity and implicit nuclear localization, whereas KTS+ isoforms will most likely be found in the cytoplasm, even though both isoforms have been shown to shuttle between the nucleus and the cytoplasm [73].

Confoundingly, transgenic mice lacking the 17AA insertion isoforms exhibit, surprisingly, no detectable phenotype abnormalities [85], even though the 17AA domain is known to generate transcription activation via prostate apoptosis response factor PAR4 (PAWR) interaction [86]. Still, 17AA domain splice site mutations have been reported in nephroblastomas [87], indicating some occult oncogenic function in humans. 

Beyond Frasier syndrome and the pathogenesis of nephroblastoma, germline *WT1* mutations have also been incriminated in mesangial glomerulosclerosis, gonadal dysgenesis and, albeit rarely, congenital diaphragmatic hernia and heart disease, as exemplified by the multiple human genetic syndromes caused by *WT1* hemizygosity or mutation [25]:

- WAGR syndrome—due to characteristic 11p deletions, *WT1* haploinsufficiency ensues, generating nephroblastoma (in ~70% of cases), aniridia, genital anomalies and intellectual impairment [88];
- Denys–Drash syndrome (DDS)—due to heterozygous *WT1* point variants, mainly involving exons 8 and 9 (zinc finger domain), which are dominant negative in nature (mutant WT1 protein and wild-type protein will dimerize, adversely affecting the functionality of the latter [89]), more severe phenotypes are seen: similarly, high prevalence of nephroblastoma, with an additional constant component of mesangial glomerulosclerosis, which will invariably lead to pediatric end-stage renal disease [90] and genital/gonadal anomalies.
- Meacham Winn Culler syndrome (MWCS)—less well defined genetically, with a phenotype lacking reno-urinary anomalies, characterized by congenital diaphragmatic hernia, genital ambiguity and complex congenital cardiac defects [25].

The renal modifications consequential to *WT1* mutation offer valuable insight into the function of the gene, both in the various stages of renal organogenesis and in mature renal tissue homeostasis. *WT1* mutant nephroblastomas spawn from an embryologic renal progenitor, the undifferentiated metanephric mesenchyme, through MET dysfunction, manifesting a distinctive, mainly stromal composition, with frequent heterotypic tissue components (usually muscle). On a molecular level, the aforementioned ectopic muscle formation can be explained through the reported upregulation of myogenic markers, secondary to mesenchymal *WT1* deletion [91]. Proper nephron development requires normal *WT1* function and transcriptional activation of WNT4 [92,93,94], whereas the aforementioned mesangial glomerulosclerosis hints at the WT1-dependent development and, afterward, in the mature state, homeostasis of podocytes, specialized renal cells which form the glomerular filtration barrier alongside the endothelium [25].

Physiological renal organogenesis implies a harmonious interaction between two cross-signaling intermediate mesoderm-derived components: the metanephric mesenchyme (origin of the nephrons, the renal functional units) and the ureteric buds (origin of the urinary transport system-ureter, renal pelvis, calyces and the entire collecting ductal system). Within this sequential process, WT1 expression levels will vary throughout, and a crucial step will be represented by the activation, within the mesenchyme of WNT4, a master regulator of the MET, which precedes nephron formation from the so-called cap mesenchyme [95]. Its pattern of expression during renal organogenesis highlights the *WT1* gene as a fundamental functional prerequisite in this process, an idea additionally supported by *WT1* knockout mice findings, in which ureteric bud mesenchymal invasion fails to occur, leading to mesenchymal apoptotic degeneration [79]. Normally, after the initial ureteric bud mesenchymal invasion, *WT1* transcription is firstly triggered in the invaded, immature, metanephric mesenchyme and becomes apparent and constantly, but scarcely, detectable, with WT1 being a prerequisite for mesenchymal viability. After mesenchymal condensation around the ureteric bud, preparation for MET ensues, with levels of both WT1, which is mandatory for MET initiation, and its target WNT4, the main driver of MET, increasing dramatically and remaining abundant throughout subsequent segmented nephron formation. Gradually, WT1 expression will become restricted to the glomerular progenitor (proximal 1/2 of the S-shaped body), and, eventually, to just the podocytes derived from this structure, as the developmental process comes to an end and the final nephron architecture (glomerulus, proximal and distal tubules, loop of Henle) emerges [25]. The highest level of WT1 expression throughout renal organogenesis is encountered in the presumptive, differentiating and mature podocytes; this is the reason why we have chosen this cell line as a positive control in the current study. In the case of connecting tubules, which form at the border between mesenchymal and ureteric bud derived structures, interconnecting nephrons and collecting ducts, the embryological origin of the constituting cell lines is unclear and controversial. Our findings of occasional low-to-moderate-intensity nuclear WT1 staining of connecting tubule cells apparently suggest, although far-fetched, that there may be a mesenchymal origin involved.

The pediatric mesangial glomerulosclerosis (in DDS/Frasier syndrome) can be explained through later-stage developmental issues regarding embryological podocyte cell line differentiation and metabolic homeostasis in adult life, with multiple investigations making it abundantly clear that *WT1* is essential for both of these processes [29,82,84,91,96]. In the current study, not only did the tumor-adjacent normal renal tissue constantly manifest for all evaluated cases, nuclear WT1 staining of the podocytes and the epithelial cells, constituting the parietal layer of Bowman’s capsule (Figure 2A), but we also found that podocytes maintained intense WT1 immunoreactivity, even in the case of renal corpuscles, which otherwise showed obvious morphological signs of degeneration (Figure 3b), being, apparently, the most resilient cell line involved in the renal blood-filtration barrier, which seemingly maintains/overexpresses WT1 in the face of cellular distress.

Developing vertebrate organisms comprise, among other things, two main cellular components: polarized epithelial cells and more mobile, non-polarized mesenchymal cells. Between these two components, a constant equilibrium must be maintained for the adequate molecular dynamics of harmonious organogenesis, and of adult organ homeostasis, for that matter. This equilibrium is maintained by vital, precisely timed waves of EMT and MET, processes which allow for the conversion of one cell line into the other [25]. If, for harmonious urogenital organogenesis, *WT1* is essential due to key roles played in the process of MET, in cardiac and diaphragmatic organogenesis, the reverse process of EMT is also *WT1*-dependent, as indicated by MWCS phenotypes.

During cardiac organogenesis, *WT1* transcription will also occur in an organized, restricted pattern, mostly in the cardiac mesothelial lining (the epicardium). At this level, through EMT (or, better said, mesothelial–mesenchymal transition—MMT), progenitors for coronary vasculature will arise (mainly for parietal smooth muscle, but also for around a fifth of embryonic endothelial cells) [97]. Indeed, *WT1* null mice will demonstrate greatly diminished ventricular functional reserve, epicardium thickness and coronary vasculature [79]. Specific epicardial *WT1* inactivation leads to embryonic demise at ~16.5 days, due to severely depleted coronary vasculature. The epicardium remains mostly intact morphologically, yet EMT/MMT is drastically diminished, as WT1-dependent transcriptional activation of SNAI1 (the main effector of EMT initiation) and repression of the fundamental epithelial cell adhesion molecule E-cadherin (Cdh1) [98] will no longer occur. Additionally, WT1 directly and indirectly regulates EMT via interactions with the WNT and retinoic acid signaling pathways [99,100]. Moreover, WT1 also facilitates epicardial cell mobility and myocardium development via direct and indirect repression of the inhibitory chemokines CXCL10 and CCL5 [101].

Beyond cardiac organogenesis, similar constant cellular components that are apparently trapped in an intermediary state, manifesting both epithelial (cell polarization and adherens junctions) and mesenchymal (abundant expression of mesenchyme-specific markers, including vimentin) traits, have been described in the mesothelial lining of other developing organs [29,102]. These cells represent a substrate of mesenchymal precursors for a wide array of specialized cellular subtypes, including fibroblasts, within various tissues [25]. Both gastrointestinal and pulmonary vasculature develop with the contribution of mesothelial precursors for parietal arterial smooth muscle [103,104]. The lung mesothelium will, additionally, also give rise to endothelial cellular precursors and will represent the substrate for airway development, spawning the muscle and cartilage of the tracheobronchial tree [105]. WT1-positive hepatic mesothelium gives rise to a specific population of hepatic stellate cells that is heavily involved in tissulary fibrosis [106]. The interstitial cells of Cajal, the intestinal pacemaker cells, are also derived from WT1-positive gut mesothelium [107]. With this in mind, similar to other connective tissue cell types, fibroblasts are derived from primitive mesenchyme and may be spawned through EMT or, conversely, represent a substrate for MET. Thus, our findings regarding the WT1 nuclear staining of stromal cells that are morphologically suggestive of fibroblasts and/or myofibroblasts in the extrarenal connective tissue, located at a significant distance from the WT1-negative tumor tissue, in 8.92% of evaluated cases is relatively unsurprising, even though the metabolic significance of this finding is unclear.

Besides the crucial roles *WT1* plays during organogenesis, it has recently become apparent that this gene maintains functionality in adulthood and has important implications regarding adult disease. In adult rodent models, WT1 expression is extremely restricted (renal podocytes, structural gonadic cells, mesothelium of various organs—the aforementioned intermediary cells—and in ~1% of immature hematopoietic bone marrow cells). Even so, the induction of a ubiquitous *WT1* deletion in adult mice (at 6 weeks) had unexpected and devastating consequences: the demise of all specimens after ∼10 days. Phenotypes showed severe glomerulosclerosis, with podocyte dedifferentiation, splenic and exocrine pancreatic atrophy, and rampant depletion fat deposits and bony tissue [108]. Despite lingering uncertainties regarding molecular pathogenesis, these dramatic phenotypes highlight the importance of WT1-dependent signaling pathways in adulthood, be they systemic, local paracrine or cell-autonomous, and their myriad of downstream effectors in maintaining global homeostasis [29] and as possible therapeutic targets.

Importantly, it seems that *WT1* transcription induction represents an inherent component of adaptive hypoxic tissulary responses. Compelling emerging evidence demonstrating direct *WT1* transcriptional activation by hypoxia-inducible factor 1 (HIF1) supports the notion of a physiological WT1-dependent adaptive mechanism to ischemia [109], while also bearing important implications regarding RCC pathogenesis. Even more compelling is the fact that, following cardiac ischemia in adults, *WT1* transcription reactivation occurs in the mature epicardium, determining epicardial cell proliferation and spawning a new wave of progenitors which have the potential to induce coronary neoangiogenesis and, albeit controversially, cellular repopulation of the myocardium [110]. Moreover, coronary vessel, post-ischemic, *WT1* transcriptional activation, has been already established [111]. Therefore, our findings, regarding intratumoral WT1 nuclear staining of endothelial cells (2 arteries and 1 vein), in otherwise WT1-negative aRCCs, may be reflective of molecular vascular adaptations to ischemia in the RCC microenvironment or the proangiogenic function of WT1 in cancers. 

Actually, in the oncological setting, for a variety of adult epithelial neoplasms, WT1 immunoreactivity has more often been documented in vascular and stromal tumor components, as opposed to the actual epithelial-derived tumor cells [25]. In contrast, in the current investigation, we report the highest percentage of exclusively nuclear WT1-positive aRCC tumor cells to date (12.5%), with a much lower rate (5.35%) of cases showing isolated endothelial WT1 nuclear immunoreactivity of intratumoral blood vessels. Conversely, corroborating the results of the current investigation, melanoma and lung cancer xenograft models express WT1 immunoreactivity at the level of tumor invading stromal and vascular elements, but not at the level of similar proximal tumor-adjacent blood vessels/stroma [25]. Promisingly, the impaired development and dissemination of new subcutaneously implanted tumors, as well as regression of already implanted and developed tumors, has been achieved by using targeted host *WT1* deletion (endothelial, hematopoietic and myeloid suppressor cells) in *Tie2-Cre* transgenic rodent models [112].

In fact, RCC represents an intensely immunogenic and angiogenic cancer, with an extremely diverse and very dynamic tumor microenvironment (TME), comprising cellular elements, namely (myo)fibroblasts, adipocytes, neuroendocrine, immuno-inflammatory and endothelial cells, dispersed within a biologically active extracellular matrix. Tumor cells interact with stromal and immune elements, both directly, by releasing various bioactive modulatory factors, acting in auto/paracrine manner, and indirectly, through hypoxic and necrotic proliferation-related events. This elaborate interplay constantly shapes and redefines the TME, with the resulting heterogeneity being considered defining and intimately linked to disease initiation, progression and therapy resistance [113]. Essentially, cancer occurs when cells evade immune destruction and proliferate, inferring an imbalance within TME immune activity between antitumor responses and tumor-promoting inflammation. Sustained efforts in evaluating inflammatory pathways associated with RCC, mainly focusing on the Von Hippel-Lindau (VHL), mechanistic target of rapamycin (mTOR), tumor necrosis factor (TNF) and STAT pathways [114], have already provided significant insight into oncogenesis and allowed for the development of efficient targeted RCC treatment. The Cancer Genome Atlas (TCGA), a large-scale coordinated sequencing effort to accelerate the molecular characterization of cancers, produced vast amounts of data. Beyond the genomic characterization of RCC [115] and identification of signature mutations exclusive to RCC, affecting the *VHL* (52%) and *PBRM1* (33%) genes [116], these data allowed for a comprehensive evaluation of associations among immune-, inflammation- and RCC-related genes [117]. 

It is worth noting that WT1 expression and protein localization are seemingly modulated by inflammation. Inflammation involves triggered immune cell accumulation and abundant release of mediators, with implicit activation of various transcription factors, such as nuclear factor-κB (NF-κB) and signal transducer and activator of transcription 3 (STAT3), to drive inflammatory signaling pathways, facilitating tissue destruction to ensure host defense [114]. Activation of NF-κB seemingly determines WT1 overexpression [118]. Conversely, in a murine podocyte injury model, rapid upregulation of the early growth response-1 (Egr-1) transcription factor apparently structurally antagonized WT1, promoting podocytes damage [119]. Moreover, post-translational phosphorylation regulates transcription factor activity, as demonstrated by the in vitro abolishment of WT1 DNA-binding activity, after phosphorylation of C-terminus zinc finger domain situses, Ser-365 and Ser393, by protein kinase (PK) A and C, respectively [120]. Nuclear localization of WT1 protein requires zinc fingers to contain two functionally independent targeting signals [121], which are altered by WT1 post-translational phosphorylation, meaning WT1 protein becomes confined to the cytoplasm [122]. In lung cancer cell cultures, TNFα induced similar WT1 cytoplasmic translocation through PKA-dependent phosphorylation and determined the overexpression of protein matrix metalloproteinase-9, which is normally silenced by WT1 [123]. Lastly, in a lipopolysaccharide murine sepsis model, renal expression of WT1/nephrin was decreased, inducing renal damage. Contrastingly, in control renal explants exposed to the pro-inflammatory cytokines TNFα and IL1β, WT1/nephrin was overexpressed [124], corroborating the notion that WT1 expression is inflammation dependent. 

Abundant expression of WT1 proteic transcripts has been noted in hematological malignancies (the bulk of acute myeloid/lymphoid leukemias) and in almost every subtype of solid adult tumor. Seeing as *WT1* has been extensively documented to be involved in the early stages of physiological hematopoiesis, being involved in triggering primitive progenitor lineage differentiation [125], overexpression in leukemias in unsurprising, yet WT1 expression has been documented in tumors which originate from tissue in which *WT1* is not physiologically expressed, implying a *WT1* oncogene function in these neoplasms. So far, *WT1* has been incriminated as a necessary contributor to KRAS-driven oncogenesis, demonstrating decisive regulatory roles regarding tumor cell responses (i.e., proliferation versus senescence) downstream of KRAS oncogenic signaling. In the murine models of KRAS-dependent lung cancer, *WT1* deletion/suppression led to senescence only in KRAS-expressing cells, and did not impact wild-type cells, while *WT1* loss determined reduction in tumor burden. Similarly, human KRAS-dependent lung cancer cell lines respond to *WT1* loss by halting proliferation and undergoing senescence [31,126]. Regardless, WT1 protein overexpression has not yet been clearly characterized as a causal contributor to carcinogenesis or a mere byproduct of it. Even so, WT1 is undeniably a promising tumor-associated antigen, with potentially revolutionary clinical applications (evaluation of prognosis, detection of minimal residual disease/relapse and immunotherapy) which are currently under investigation.

Multiple human cancer cell culture experiments, derived from both leukemias and solid neoplasms, have shown that neutralization of *WT1* via targeted antisense oligomers [127,128] and small interfering (si)RNA [129] abolished further tumor cell division. In *WT1*-transgenic mice with forced hematopoietic progenitor WT1 overexpression, rapid onset acute myeloid leukemia was induced by AML1-ETO fusion transduction, as opposed to the wild-type model, where transduction was insufficient to cause systemic disease [130]. Furthermore, in cell cultures (hematopoietic, immune and neoplastic), WT1 overexpression has been shown to block cellular differentiation, yet facilitate proliferation [131,132,133] and mobility [134], while hindering apoptosis [135]. Thus, it seems that WT1 may not only facilitate tumor dissemination, but also cancer resilience, increasing tumor cell survival through its anti-apoptotic function. Actually, an emerging chemotherapy-resistance mechanism, driven by WT1 downregulation, due to transcript cleavage via HTRA2, a protease overexpressed under cytotoxic therapy, has been shown to increase cancer cell survival, based on C-MYC/JUNB upregulation, which is normally repressed by WT1 [136]. Recently, a natural splice variant of wild-type *WT1*, the Ex4a(+)WT1 isoform, which is missing the whole zinc finger domain (truncated C-terminus), has been identified and may hold therapeutic value, as it has been shown to decrease WT1 major isoform anti-apoptotic function. Ex4a(+)WT1 isoform shows minor endogenous expression in myeloid leukemia/solid tumors cells, but during apoptosis, it becomes overexpressed, in spite of major isoform depletion. Ex4a(+)WT1 overexpression will block major isoform transcriptional activation of the mitochondrial anti-apoptotic protein Bcl-xL, thus inducing mitochondrial damage/apoptosis, while Ex4a(+)WT1 siRNA neutralization halts apoptosis [137].

Beyond its established roles in organogenesis and adult tissue maintenance and repair, EMT is emerging as an essential process in tumor pathogenesis, proliferation and dissemination [138], and it is potentially involved in aRCC pathogenesis [139,140,141]. One proposed pathogenesis model for aRCC states that the developmental and physiological processes of MET, which is responsible for mesenchymal differentiation into mature renal tissue, is hijacked and becomes reversed. Thus, aRCC occurs through EMT-induced dedifferentiation of renal cells [139]. Predictive EMT gene signatures have been investigated and statistically validated, independently or against extensive TCGA data sets. For example, a set of genes characterizing normal tissulary repair response via quiescent fibroblast activation indicative of EMT has been shown to associate poor prognosis with high mortality when expressed in ccRCC [140]. Recently, a differential gene expression investigation focusing on EMT and cell cycle proliferation (CCP) pathways established and validated against a large TCGA cohort a novel EMT score for risk stratification in ccRCC. The simultaneous activation of both EMT and CCP pathways showed the worse prognosis. The TCGA WT1-overexpression ccRCC cohort consistently showed enrichment of both pathways [142]. 

These genetic profiling efforts will eventually pinpoint key genetic targets, which will translate into RCC-associated proteins of interest, facilitating the development of better clinical evaluations for risk stratification and novel targeted therapeutics. An automated quantitative immunofluorescence analysis that exemplified this clinical translation assessed EMT-associated protein (WT1, SNAIL, SLUG, E-cadherin and phospho-β-catenin) expression in 61 patients with ccRCC. Despite successful, accurate detection of all antigens in ccRCC specimens, only SLUG and SNAIL were able to adequately stratify patients into high- and low-risk progression-free survival groups. The small cohort evaluated, as well as inherent protein expression quantification limitations (a since discontinued polyclonal C-terminus targeted WT1 antibody, GTX15249, was used), may account for the lack of statistical validation of prognostic value for the other antigens evaluated [143].

Immunotherapy, the fourth type of cancer therapy after surgery, chemotherapy and radiotherapy, is an emerging and promising field, which may hold the key to a definitive cure for cancer, seeing as it is the only oncotherapy capable of targeting and annihilating non-dividing quiescent cancer stem cells [144]. With amounting data corroborating WT1 oncogene activity in leukemo/tumorigenesis, WT1-targeted immunotherapy applications were soon to follow. Actually, there are quite a few inherent advantages to using WT1 as a tumor-associated antigen target, namely abundant expression in a great variety of neoplasms, with highly selective physiological mature tissue expression and a wide gap in expression levels between healthy and neoplastic tissues, but also an oncogenic potential born of its intrinsic activity, which suggests robustness against emergence of escape variants [144]. WT1 is also highly immunogenic, as both peptide immunization [145,146,147,148,149,150] and DNA-based immunization [151] were shown to be effective in inducing WT1-specific cytotoxic T lymphocytes (CTLs) capable of more easily identifying and destroying WT1 positive tumor cells. Furthermore, as seen in the case of hematopoietic malignancies, patients will often naturally develop WT1-targeted immune responses, even anti-WT1 IgG antibodies [152]; however, in spite of these aberrant immune responses targeting an intrinsic self-protein, CTLs did not exhibit any self-destructive activities [144]. In mouse models, even after extensive immunization (optimally activated antigen-presenting cells displaying WT1 peptide), no signs of immune self-aggression were seen [149], and, even more encouragingly, self-aggressive responses have not yet been observed in any of the human trials, either [153]. Conversely, in the ongoing WT1 peptide-based immunotherapy phase I/II trials, frequent clinical responses and even drastic tumor regression have been reported for leukemia [153], myelodysplastic syndrome [154] and solid tumors, such as lung [153,155] and breast cancers [153,156].

In 2007, within a larger multicenter phase I/II clinical trial on WT1-based immunotherapy, the first report of WT1 peptide vaccination in advanced RCC was published. After the HLA serotype was confirmed, three patients with metastatic ccRCC were vaccinated weekly with a HLA-A*24:02-binding WT1 peptide, for 3 months. Tumor development was halted, and disease stability was achieved in two cases, and, importantly, no new metastases appeared in these patients for a prolonged period, with no harmful side effects being observed [144]. More recently, vaccination with WT1 peptide-loaded dendritic cells (DCs), coupled with targeted immunotherapy and/or traditional chemotherapy, was investigated in five RCC and five bladder cancer patients with relapsed or refractory disease. No vaccination-related severe adverse events were observed, with seven patients maintaining durable disease stability and three other patients experiencing progression. The method seems beneficial, safe and feasible for patients in an advanced stage of RCC or bladder cancer [41].

Most recently, WT1-specific CTLs were regenerated from allogeneic homozygous human leukocyte antigen (HLA) haplotype–induced pluripotent stem cells (iPSCs) and have shown therapeutic efficacy in a patient-derived RCC xenograft tumor model [42]. Initially, CTLs were regenerated from HLA haplotype-homozygous iPSCs and then transduced, using a viral vector (Lentivirus), with WT1-specific T-cell receptor (TCR) α/β genes, which had already undergone clinical validation [157]. For the treatment model, RCC-patient-derived xenografts were obtained, both WT1 positive, but also WT1 negative (serving as a specificity control), and inoculated subcutaneously into the back of a *NOG* mouse, on the right side and left side, respectively. During the following 30 days, 12 intraperitoneal injections of regenerated WT1-TCR-CTLs (1 × 10^7^ cells) were administered. Tumor development impairment was evident macroscopically in the WT1-positive tumors, while no response was seen in the WT1-negative tumors. Histological evaluation showed a significantly higher amount of CD8 T-cell infiltration in WT1+ versus WT1− tumors. Interestingly, for the RCC-patient-derived xenograft model, investigators analyzed a total of 16 resected RCC specimens for WT1 antigen expression, using the 6F-H2 monoclonal antibody, and found WT1 positivity (not mentioned if nuclear or cytoplasmic) in 13 (81.25%) cases [42], the highest reported so far.

Beyond the burning need for further molecular characterization of key genes involved in oncogenesis, as we imminently enter into a new era of personalized, targeted cancer immunotherapy, adequate case selection will surely prove to be vital. In the case of RCCs, standardization of protein expression quantification, not only for WT1, but other tumor-associated antigens (CTLA4, PD1/PDL1) as well, is mandatory for further progress and will require extensive characterization of staining patterns, with both specificity and sensitivity, for the plethora of commercially available IHC antibodies, on larger RCC populations and for individual RCC subtypes.

Indeed, the wide reported variability of WT1 protein expression levels and IHC staining patterns may be a result of gene complexity and particular metabolomics of individual isoforms, as well as heterogeneous definitions of WT1-positivity, the myriad of available antibodies and the different staining methodologies used, not to mention the various N-terminal truncated isoforms [158,159,160] and the recent C-terminal truncated variant [137], which complicate WT1 transcript evaluation even further. One investigation, a comparative study between WT1 IHC antibodies, used the newer clone WT49 and the conventional clone 6F-H2 to stain 40 malignant pleural mesotheliomas, 55 lung carcinomas and 10 intrathoracic synovial sarcomas [161], and their results corroborate with ours. Within the malignant pleural mesothelioma population, there were discrepancies between positive cases, depending on the antibody used: 30(75.0%) positive cases with clone WT49 and only 26 (65.0%) with 6F-H2. Clone WT49 showed nuclear WT1 immunoreactivity in four (7.2%) lung carcinomas and in one (10.0%) synovial sarcoma, while clone 6F-H2 showed no nuclear immunoreactivity in any of the tumors, except for malignant pleural mesotheliomas. More importantly, no cytoplasmic WT1 immunoreactivity was seen in any of the tumors evaluated by using clone WT49, whereas clone 6F-H2 showed WT1 cytoplasmic staining in 7 (17.5%) malignant pleural mesotheliomas, 17 (30.1%) lung carcinomas, and 5 (50.0%) synovial sarcomas [161]. In line with these findings, in the current study, we used clone WT49 on an aRCC population and obtained the same exclusively nuclear staining pattern. 

## 5. Conclusions

All in all, *WT1* is a complex gene with a seemingly endless myriad of transcript isoforms, acting upon and being acted upon by equally complex molecular signaling pathways. The data regarding WT1 protein expression in aRCCs are quite limited and highly inconsistent. Multiple IHC antibodies, targeting different areas of WT1 protein (C- or N-terminus), have shown different immunoreactivity rates and staining patterns. In this study, we used, for the first time, the recent N-terminus targeted, WT1 IHC antibody, clone WT49, to evaluate WT1 protein expression in the largest aRCCs cohort to date. Despite including cytoplasmic staining in the definition of positivity for WT1 immunoreactivity, as well as previous IHC findings in RCCs, using other N-terminus targeted WT1 antibodies, we exclusively reported on nuclear WT1 staining with clone WT49 and the highest rate of nuclear WT1-positive aRCCs so far, 12.5%. Multiple molecular interactions influence WT1 protein expression and localization. For now, WT1 protein expression in aRCCs is insufficiently investigated. Additional, comparative IHC analyses of the many commercially available antibodies are essential in order to standardize the quantification of WT1 protein expression in aRCC and its many subtypes. WT1-targeted RCC immunotherapy applications have shown very promising results, but they will require improved protocols for patient selection, as clinical trials are underway. Even so, aRCC remains, to date, the most lethal genitourinary cancer. Moving forward, the investigation of signaling pathways driving carcinogenesis and progression, as well as the elaboration of adequate clinical tools for risk stratification, will be vital for improving aRCC management and outcomes.

## Figures and Tables

**Table 1:** Modified ISUP/WHO Fuhrman nuclear grading system adapted from Moch et al. [4].

Grade	Characteristics
1	Absent or inconspicuous nucleoli and basophilic under 400× magnification.
2	Conspicuous and eosinophilic nucleoli under 400× magnification, but not prominent under 100× magnification.
3	Eosinophilic and prominent nucleoli under 100× magnification.
4	Extreme nuclear pleomorphism and/or giant neoplastic cells and/or any degree of sarcomatoid and/or rhabdoid dedifferentiation.

**Table 2:** WT1 quantitative expression protocol.

Grade	Definition
0	Absent WT1 staining.
+1	Rare positive nuclei/cytoplasm, constituting < 2% of total tumor cell population.
+2	Positive nuclei/cytoplasm constitute < 10% of total tumor cell population.
+3	Positive nuclei/cytoplasm constitute > 10% of total tumor cell population.

**Table 3:** Positive intratumoral WT1 staining cases.

Sex	Age	Subtype	Nuclear Grading	TNM	Quantitative WT1	Qualitative WT1	Clinical Outcome
Male	68	Clear cell RCC	2	pT2b	+1	weak/ moderate	Died 3 years later after 2 lines of treatment for pulmonary metastases.
Male	70	Clear cell RCC	2	pT2a	+3	moderate	No recurrence at 5 years.
Male	65	Clear cell RCC	1	pT1a	+2	high	No recurrence at 5 years.
Female	58	Clear cell RCC	1	pT1a	+2	high	No recurrence at 5 years.
Male	74	Clear cell RCC	2	pT1b	+1	high	Died of cardiovascular event, but no recurrence at 4 years.
Male	62	Clear cell RCC	2	pT3a	+2	high	Solitary pulmonary metastasis after 2.5 years, treated, no recurrence at 5 years.
Male	68	Clear cell RCC	1	pT1b	+3	moderate	No recurrence at 5 years.

## Data Availability

Our data are available at https://data.mendeley.com/datasets/v42wd7xsxj/1 (accessed on 29 December 2021).
